# A phase II study of S-1 monotherapy administered for 2 weeks of a 3-week cycle in advanced gastric cancer patients with poor performance status

**DOI:** 10.1038/sj.bjc.6603902

**Published:** 2007-07-24

**Authors:** H-C Jeung, S Y Rha, S J Shin, J B Ahn, S H Noh, J K Roh, H C Chung

**Affiliations:** 1 Cancer Metastasis Research Center, Yonsei Cancer Center, Yonsei University College of Medicine, Seoul, Korea; 2 Department of Internal Medicine, Yonsei University College of Medicine, Seoul, Korea; 3 Department of Surgery, Yonsei University College of Medicine, Seoul, Korea

**Keywords:** S-1, gastric adenocarcinoma, performance status

## Abstract

Systemic chemotherapy for gastric cancer is often associated with treatment-related toxicity, which is particularly severe in patients with a poor performance status. In this paper, we describe the first study to evaluate S-1 monotherapy as an option for advanced gastric cancer patients who are not candidates for combination chemotherapy due to poor clinical condition. Fifty-two patients with Eastern Cooperative Oncology Group (ECOG) performance scale 2–3, whose general condition had made use of combination chemotherapy impossible, were enrolled. S-1 was administered to 30 patients as second- or third-line therapy. The initial dose of S-1 was 35 mg m^−2^, administered b.i.d for 14 days every 3 weeks. With a median follow-up period of 33 weeks, the median progression-free survival, and overall survival were 11 weeks (95% CI, 8–14) and 33 weeks (95% CI, 19–47), respectively. The overall 1-year survival rate was 29% by intent-to-treat analysis. The overall response rate was 12% (95% CI, 3–21), and the percentage of stable disease was 35%, resulting in the disease control rate of 47% (95% CI, 32–60). Significant drug-related toxicity included grade 3 diarrhoea (14%), anorexia (14%), fatigue (10%), neutropenia (10%), and leucopenia (6%). In conclusion, this study indicated the modest activity of S-1 in gastric cancer patients with poor performance status.

Performance status is a widely accepted parameter that predicts response to chemotherapy and survival, and it is associated with many factors such as age, previous chemotherapy, and comorbidity (Lavin *et al*, 1982; Janunger *et al*, 2001; Wilson *et al*, 2005). In advanced gastric cancer, patients are likely to suffer from poor general condition due to anorexia and weight loss, often as a consequence of peritoneal carcinomatosis. These patients have been excluded from clinical trials, and chemotherapeutic options for them are quite limited (Cunningham *et al*, 1987; Hsu *et al*, 1997).

Combination chemotherapy is a standard approach in advanced gastric cancer, with many combination regimens showing a response rate of 35–45% for first-line treatment. However, these regimens are inevitably accompanied by substantial toxicities, which reduce their value as a palliative treatment. This toxicity is particularly significant in patients whose performance status is compromised. S-1 is a fourth-generation oral fluoropyrimidine that was developed to mimic protracted continuous infusion of 5-fluorouracil (5-FU). In the phase II trials conducted in Japan, S-1 monotherapy demonstrated promising activity which was comparable to combination chemotherapy in advanced gastric cancer, and it has been one of the preferred agents for gastric cancer (Sakata *et al*, 1998; Sugimachi *et al*, 1999; Koizumi *et al*, 2000). Moreover, it has a safety profile, which is favourable compared with other oral fluoropyrimidines used in gastric cancer. In addition, single agent 5-FU has been an option for patients with poor general condition, and also for patients complicated with disseminated intravascular coagulation (Chao *et al*, 2000).

Based on these observations, S-1 may be a substitute for the conventional chemotherapy in patients with poor general condition. We conducted the first prospective study to evaluate the feasibility of S-1 as an option for gastric cancer patients who were not candidates for more intensive chemotherapy due to poor clinical condition.

## PATIENTS AND METHODS

### Patient eligibility

This study was designed as a single-institutional phase II trial. Patients with histologically proven metastatic and/or relapsed gastric adenocarcinoma were considered eligible for the study when they met all of the following criteria: aged ⩾18; Eastern Cooperative Oncology Group (ECOG) performance scale 2–3; assessable disease with or without measurable lesion; either chemotherapy-naïve or having completed chemotherapy due to disease progression within 3 months before entry; and adequate haematological, renal, and hepatic functions. The latter was defined as neutrophil ⩾1500 *μ*l^−1^, platelet ⩾75 000 *μ*l^−1^, serum creatinine ⩽1.5 mg dl^−1^, total bilirubin ⩽1.25 (or 1.5) × upper limit of normal (ULN), and serum transaminases ⩽2.5 (or 5.0) × ULN in the absence (or presence) of liver metastasis. Patients were excluded from the study if they had concurrent cancer within the past 3 years (excluding basal cell carcinoma of the skin or cervical carcinoma *in situ*), active metastasis to central nervous system, or uncontrolled significant comorbid conditions. After the protocol was approved by the institutional review board, written informed consent with ICH Guidelines was obtained from patients.

### Treatment schedule

The starting dose of S-1 was 35 mg m^−2^ twice daily (b.i.d). S-1 was administered within 1 h after the morning and evening meals for 14 consecutive days, followed by a 7-day resting period. The S-1 dosage assigned to the patients was calculated based on body surface area, which was different from the Japanese dosing system. The planned dose intensity was 327 mg m^−2^ week^−1^. The schedule was repeated until the occurrence of disease progression, unacceptable toxicities, or patient's refusal. In the absence of evidence of disease progression, the patients were allowed to continue S-1 treatment to the maximum 12 cycles. A dose reduction of 10 mg m^−2^ a day was made if ⩾grade 3 haematological or non-haematological toxicity was shown in the previous cycle. Dose re-escalation was not allowed. Patients who required more than 4 weeks of rest for recovery from any toxicity other than alopecia, nausea, vomiting, or anaemia, or who required dose reduction of more than two steps (total 20 mg m^−2^ a day) were withdrawn from the study.

### Evaluation of response and toxicity

Weight loss, comorbidity, and performance status at presentation were established by direct questioning of the patient during a preliminary assessment by a physician at the first attendance for S-1 treatment. Baseline evaluations of each patient included complete medical history with physical examination, complete blood count (CBC), serum chemistry, urine analysis, and electrocardiography. A radiological evaluation was completed within 3 weeks before treatment. Fibre-optic gastroduodenoscopy and positron-emission tomography were planned to examine complete response (CR) of all measurable lesions. During treatment, patients were evaluated by a weekly CBC. A physical examination including weight, performance status, and serum chemistry were performed before each subsequent cycle. Radiological studies were repeated every two cycles.

Treatment response was evaluated by spiral CT scan according to the guidelines of the Response Evaluation Criteria in Solid Tumors (RECIST) committee. The response was analysed according to an intent-to-treat (ITT) analysis. A measurable lesion was defined as 10 mm in the longest dimension. If a patient was documented as having a CR or a partial response (PR), the response was confirmed at least 4 weeks after the first evidence of response.

Progression-free survival (PFS) was defined as the time elapsed from the start of treatment until disease progression or death of any cause, and overall survival (OS) was defined as the start of treatment to death. Time-to-treatment-failure was defined as the time interval between treatment initiation and cessation due to any reason. All patients were evaluated for toxicity from the time of their first cycle. Toxicity was evaluated as a grade according to the NCI-CTC (version 2.0).

### Biostatistics

The primary aim of this study was to test the hypothesis that 1-year survival would improve by what degree compared to historical controls. The study was designed to have an 80% power to show an improvement in 1-year survival rate to 30% from historical control of 15 with 5% type-I error, using two-sided testing, and assuming exponential overall survival times. According to Minimax phase II design, a sample size of 48 patients was required. Considering a 5% drop-out rate, 52 patients were needed for this trial. The secondary aims included response rate in patients according to RECIST criteria, safety, PFS, time-to-treatment-failure, and OS. Time-dependent variables were analysed using the Kaplan–Meier method and compared using the log–rank test. Multivariate analysis was performed using Cox's proportional hazard regression model. Exact 95% confidence interval (CI) was provided for proportions.

## RESULTS

### Patient characteristics

A total of 52 patients were enrolled between August 2004 and July 2006. Baseline patient characteristics are provided in Table 1. All patients were evaluable for survival. The median age was 61 years, including 12 patients (23%) who were ⩾70 years old. Twenty-four patients (46%) had performance status of grade 3. Median value of weight loss from the diagnosis of gastric cancer to S-1 start was 7% (range 5–23%) and 21 patients (40%) suffered more than 10% weight loss. Twenty-six patients had comorbidity other than gastric cancer. More than half of the patients received prior first- or second-line palliative chemotherapy. Twenty-two patients (42%) had no history of palliative chemotherapy. Site of metastasis commonly included peritoneum (45%), abdominal lymph nodes (33%), and stomach (33%).

Table 2 summarises the prior chemotherapy which our patients received. Twenty-one patients (40%) received prior adjuvant chemotherapy after curative resection. The median time elapsed from documentation of disease progression (or relapse) of previous regimen to S-1 treatment was 36 days (range 14–73). The median number of cycles of previous chemotherapy was eight (range 2–24) per patient.

### Treatment outcomes

The median dose administered per day was 100 mg (range 80–130). A total of 233 treatment cycles (median 2.5, range 1–12) were administered. Six patients (12%) completed the planned 12 cycles. Four patients were subjected to dose reduction by one step, and two patients were by two steps due to grade 3 adverse events. Total 31 cycles were delayed in 16 patients with the median delayed duration of 1 week (range 1–4). The reason of delay was as follows: non-haematological toxicity in 13 cycles; haematological toxicity in 10 cycles; and the patients' will in the remaining eight cycles. Finally, the median dose intensity of all patients was 308 mg m^−2^ week^−1^ (range 163–327), which the relative dose intensity was 94%. Eighteen patients (35%) were transferred to the other chemotherapy regimens after disease progression was documented.

### Survival

**Figure 1:**
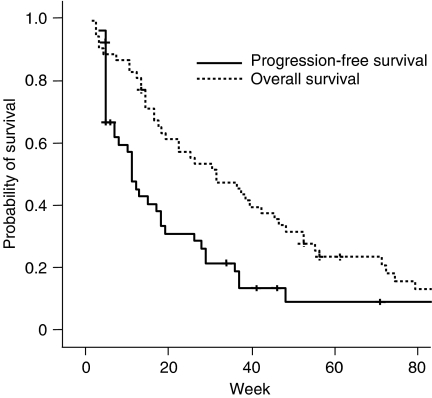
Survival analysis of all patients (*N*=52).

At the time of analysis, all the patients finished S-1 treatment. With the median follow-up period of 33 weeks (range 3–98), 46 patients showed disease progression, and all but eight patients (85%) expired. Median overall survival duration was 33 weeks (95% CI, 19–47), and the 1-year survival rate was 29%. Median PFS was 11 weeks (95% CI, 8–14) (Figure 1). Patients with measurable disease (*n*=30) demonstrated a median PFS of 10 weeks (95% CI, 2–17) while patients with assessable, but non-measurable disease (*n*=22) had a median PFS of 19 weeks (95% CI, 5–33) (*P*=0.12).

When the PFS was analysed with respect to prior chemotherapy, among patients who received S-1 as first-line treatment the median PFS was 18 weeks (95% CI, 7–23), whereas second- or third-line patients showed 8 weeks (95% CI, 1–15) (*P*=0.07). Median time-to-treatment-failure of all the patients was 9 weeks (95% CI, 14–104).

When we compared the PFS profile according to clinical parameters by multivariate analysis, patients' age (>65) (*P*=0.015), and previous exposure to chemotherapy (*P*=0.018) were the significant factors for poor PFS for measurable disease. For non-measurable disease, no factor was found to be significant for PFS. Severe weight loss (grade >2) (*P*=0.008), male sex (*P*=0.02), previous exposure to chemotherapy (*P*=0.012), and prior gastrectomy (*P*=0.03) were independent factors for poor overall survival.

### Response

Response to therapy was assessable in all but one patient, who withdrew the consent after the first cycle (Table 3). The overall response rate was 12% (95% CI, 3–21), and the percentage of stable disease was 35%, resulting in the disease control rate of 47% (95% CI, 32–60). Of 30 patients with measurable disease, three patients achieved a PR, and one had a CR giving an overall response rate of 13% by ITT analysis. For 22 patients with non-measurable disease, two patients showed CR, and nine patients showed non-progressive disease. All but eight patients stopped S-1 treatment because of disease progression. Forty-four patients had PD involving progression of pre-existing lesions, while the remaining patients (15%) showed new lesions: peritoneal seeding (*n*=3), lung (*n*=2), brain (*n*=1), liver (*n*=1), and bile duct (*n*=1). When the response was analysed with respect to prior chemotherapy, among patients who received S-1 as first-line treatment there were five responses (23%), whereas 30 second- or third-line patients showed only one CR without a PR (3%) (*P*=0.07). When the response was also analysed according to performance status, in ECOG two patients there were three responses (11%), and also in ECOG three patients, there were three responses (13%).

### Toxicity

Toxicity was assessable in all 52 patients (Table 4). There was one case of sudden death during treatment of an 81-year old man with a history of hypertension who received six courses of treatment. There was no evidence of disease progression or toxicity in this patient, but there occurred sudden cardiac death in the resting period of cycle 6. Common significant drug-related toxicity included grade 3 diarrhoea (14%), anorexia (14%), fatigue (10%), neutropenia (10%), and leucopenia (6%). Two patients developed febrile neutropenia (4%). Three patients suffered weight loss of more than 10% during S-1 treatment. Seven hospitalisations possibly related to treatment occurred in six patients (12%); gastrointestinal toxicity (*n*=6), and aspiration pneumonia accompanied with febrile neutropenia (*n*=1).

## DISCUSSION

To the best of our knowledge, this is the first prospective report on S-1 treatment in gastric cancer patients with poor performance status. Old age, poor performance status, comorbidity, and significant weight loss are adverse prognostic factors for gastric cancer (Andreyev *et al*, 1998; Trumper *et al*, 2006). Regimens of cisplatin, doxorubicin, or etoposide are associated with severe drug-related toxicity that could offset the potential clinical benefit. Recently developed chemotherapy regimens including taxanes, irinotecan, and oxaliplatin have better safety profiles compared to the older regimens. However, the benefit of these new regimens has not been evaluated in patients with poor performance status because such patients are traditionally excluded from clinical trials.

The definition of poor general condition differs among studies, but the first chemotherapeutic consideration for poor general condition was the ELF regimen (etoposide, 5-FU, leucovorin) (Wilke *et al*, 1990). This regimen showed favourable compliance for elderly patients or those with cardiac disease preventing doxorubicin treatment, but 20% of patients suffered from grade 3–4 neutropenia and 7% from grade 3 diarrhoea. High-dose 5-FU with leucovorin (HDFL) showed promising efficacy in patients with poor general condition of old age, performance status, comorbidity, malnutrition, and cytopenia (Hsu *et al*, 1997). However, this regimen was associated with considerable non-haematologic toxicity of mucositis and vomiting, and high HDFL-induced neurotoxicity cast another concern on its safety. A recent report dealt with FOLFIRI regimen in poor performers (Beretta *et al*, 2006). Its response rate was promising (40%), but grade 3–4 neutropenia (17%) was forcing dose reduction. Therefore, none of these regimens is widely accepted as the reference, and there is a vital need for new regimens that procure dose intensity without impeding safety.

S-1 has been shown to be safe in Korean patients. In the previous phase II trial, patients tolerated the highest dose intensity ever reported, and grade 4 toxicity was not reported (Jeung *et al*, 2007). Therefore, we hypothesised that S-1 could also be applied in patients whose general condition is compromised. When designing this trial, we adopted a 2-week schedule of S-1 instead of the conventional 4-week schedule. It is based on the knowledge that: (1) S-1-related adverse reactions commonly appear 2–3 weeks after treatment starts (Nagashima *et al*, 2005); (2) a 2-week schedule showed the possibility of mitigated toxicity and prolonged the medication period (Kimura *et al*, 2003); (3) this schedule showed feasibility in heavily pretreated metastatic colorectal cancer (Jeung *et al*, 2006); and (4) recent phase I study of 2-week S-1 schedule demonstrated an antitumour activity in chemotherapy-refractory gastric cancer, with a similar pharmacokinetic profile to the conventional schedule (Zhu *et al*, 2007).

Another concern in designing this trial was how to measure the efficacy of S-1 in our patients, since few studies have concentrated on this poor performance status population (Wilke *et al*, 1990; Hsu *et al*, 1997; Jeung *et al*, 2007). Gastric cancer is mixed with considerable non-measurable proportion, and it is higher in Korean patients compared with Western populations. Peritoneal metastasis or locoregional recurrence comprises approximately 50% of initial recurrence of gastric cancer, most of which cannot be numerically assessed by conventional imaging (Yoo *et al*, 2000). Therefore, we concentrated on 1-year survival rather than response rate to measure the clinical benefit of S-1.

Performance status is an indicator of a patient's global ability and it correlates with survival time. Preoperative performance status of ECOG 2–3 in non-curable gastric cancer patients is associated with a 1-year survival rate of 17%, compared with 43% for ECOG 0-1 patients (Maehara *et al*, 1993). In a prospective trial of 5-FU and cisplatin, ECOG 2–3 patients showed a 1-year survival of 30% (Rougier *et al*, 1994). Our study was designed to prove a 1-year survival of 30%, which is somewhat high considering the patients' general condition and proportion of prior exposure to chemotherapy. Nevertheless, this survival rate is a currently acceptable range expected from any new regimen aiming at second- or third-line treatment for gastric cancer (Assersohn *et al*, 2004; Chun *et al*, 2004; Wilson *et al*, 2005; Horinaka *et al*, 2006).

We obtained a 1-year survival rate of 29% with a response rate of 11%. This suggests an activity of S-1 in gastric cancer patients even with poor performance status. Although direct comparison is difficult, our result was comparable to other single-agent chemotherapy regimens in pretreated patients or poor performers (Cascinu *et al*, 1998; Chun *et al*, 2004; Horinaka *et al*, 2006). Moreover, there was no difference in response or treatment compliance according to other poor prognostic factors such as age, comorbidity, or performance status. However, it should be noted that treatment duration and efficacy seems dependent on previous treatment. As first-line treatment, the median treatment cycle was four, and five patients (23%) completed 12 cycles. Five patients (23%) showed an objective response, which was comparable to a conventional phase II trial of S-1 (Jeung *et al*, 2007). In contrast, S-1 in second- or third-line treatment showed only one response, and the median treatment cycle was two. This implies that although S-1 monotherapy has an activity in poor performance status, this activity appears to be restricted to first-line chemotherapy. There is currently inadequate evidence that S-1 monotherapy could be successfully used for salvage treatment in pretreated cases.

Another point to consider is that we observed a different toxicity profile than that reported in a previous phase II trial (Jeung *et al*, 2007). Our patients suffered more haematologic and non-haematologic toxicity, and the incidences of grade 3 neutropenia, diarrhoea, and anorexia were increased. It is no wonder if we assume that poor performance status reflects the global deterioration of the host-defense activity against the tumour and treatment burden. Although we did not assess the quality of life in this study, most patients (83%) did not suffer further weight loss during the S-1 treatment, and two patients gained weight and increasing appetite. Moreover, rare grade 4 toxicity and the lack of toxicity-related death indicate that S-1 could be applied safely to this clinical setting. However, our result also indicates that scrupulous evaluation of the adverse reaction needs for these patients is because of the more severe toxicity profile.

To conclude, our prospective study suggested that S-1 monotherapy is tolerable and has modest activity in gastric cancer patients with poor performance status. Owing to the natural history of gastric cancer, patients are particularly prone to clinical deterioration after diagnosis or during treatment course. Therefore, it is especially important to consider these patients to improve the global treatment outcome of gastric cancer.

## Figures and Tables

**Table 1:** Patient characteristics

**Patient characteristics**	**Number of patients (%)**
**Total enrolled**	**52**
**Evaluable**	**52**
Median age (years) (range)	61 (36–81)
Male: Female	33:19
	
*ECOG Performance status*	
2	28 (54)
3	24 (46)
	
*Primary site*	
Cardia	7 (13)
Non-cardia	44 (85)
Unknown	1 (2)
	
*Histological type*	
Well-moderate differentiated	14 (27)
Poorly differentiated	24 (46)
Signet ring cell	10 (19)
Others	4 (8)
	
*Prior gastrectomy*	
Yes	33 (64)
No	19 (36)
	
*Prior chemotherapy* a	
Yes	30 (58)
No	22 (42)
	
*Site of measurable lesion*	
Abdominal LN	17 (35)
Liver	10 (20)
Abdominal mass	9 (18)
Mediastinal LN	6 (12)
Cervical LN	4 (8)
Lung	3 (7)
	
*Site of non-measurable lesion*	
Carcinomatosis	23 (29)
Stomach	20 (25)
Bowel	15 (19)
Bone	4 (5)
Others	18 (22)
	
*Metastatic site per patient*	
1	12 (23)
2	21 (40)
⩾3	19 (37)

ECOG=Eastern Cooperative Oncology Group; LN=lymph node.

a As a palliative chemotherapy.

**Table 2:** Summary of prior chemotherapy

	**Number of patients**				**Dose intensity**	**Treatment cycle**
	**Total**	**CR-PR**	**SD**	**PD**	**Median (range)**	**Median (range)**
*Adjuvant*						
Doxorubicin	13				1.00 (0.82–1.00)	12 (5–12)
Taxanes	6				1.00 (0.73–1,00)	6 (5–9)
Cisplatin	2				NA (0.84, 1.00)^a^	NA (8, 9)^a^
						
*First-line*						
Taxanes	19	5	10	4	1.00 (0.75–1.00)	6 (2–9)
Cisplatin (±irinotecan)	5	2	3	—	0.94 (0.56–1.00)	6 (5–10)
Capecitabine	2	2	—	—	NA (1.00, 0.93)^a^	NA (7, 9)^a^
Irofulven	2	—	1	1	NA (1.00, 1.00)^a^	NA (2, 6)^a^
Oxaliplatin	2	—	2	—	NA (0.98, 0.85)^a^	NA (8, 10)^a^
						
*Second-line*						
Taxanes	7	2	3	2	0.93 (0.58–1.00)	5 (2–9)
Cisplatin (±irinotecan)	3	—	1	2	0.84 (0.49–0.89)	2 (2–6)
Capecitabine	2	—	2	—	NA (0.94, 0.95)^a^	NA (4, 9)^a^

CR=complete response; PD=progressive disease; PR=partial response; SD=stable disease.

a Median value was not obtainable due to small patient number (*N*=2).

**Table 3:** Evaluation of tumour response

	**Patients (*n*)**	**CR**	**PR**	**SD**	**PD**	**CD**	**NE**	**RR (%)**	**DCR (%)**
Overall (95% CI)	52	3	3	18	22	5	1	12 (3–21)	47 (32–60)
Measurable	30	1	3	9	16	1	—	13	43
Non-measurable	22	2	—	9	6	4	1	9	50

CD=clinical deterioration; CI=confidence interval; CR=complete response; DCR=disease control rate; NE=not evaluable; PD=progressive disease; PR=partial response; RR=response rate; SD=stable disease.

**Table 4:** Evaluation of toxicity per patient

	**Number of patients (*N*=52)**					
	**G1**	**G2**	**G3**	**G4**	**Toxicity of all grades^a^ (%)**	**Toxicity of grade 3–4 (%)**
*Haematologic toxicity*						
Anaemia	8	23	2	1	65	6
Leukopaenia	6	9	3	—	35	6
Neutropaenia	7	7	5	—	37	10
Thrombocytopaenia	4	2	—	2	15	4
						
*Non-haematologic toxicity*						
Diarrhoea	10	3	7	—	39	14
Nausea	17	10	1	—	54	2
Vomiting	8	7	1	—	32	2
Mucositis	15	4	—	—	37	—
Anorexia	15	20	7	—	81	14
Fatigue	17	13	5	—	67	10
Weight loss	4	3	—	—	14	—
Dyspepsia	15	5	1	—	40	2
Skin rash	8	1	—	—	17	—
Itching sensation	12	2	—	—	27	—
Skin pigmentation	13	1	—	—	27	—
Hand – foot syndrome	2	2	1	—	10	2
Abdominal pain	12	2	—	—	27	—
Elevated creatinine	8	—	—	—	15	—
Elevated AST/ALT	10	4	—	—	27	—
Elevated ALP	12	1	2	1	29	6
Hyperbilirubinaemia	8	2	1	1	23	4

ALP=alkaline phosphatase; ALT=alanine aminotransferase; AST=aspartate aminotransferase; G=Grade.

a Grading was according to the NCI-Common Toxicity Criteria (Version 2.0).
